# Endoscopic suturing ligation and fundoplication for proton pump inhibitor-resistant severe reflux esophagitis

**DOI:** 10.1055/a-2571-5803

**Published:** 2025-04-11

**Authors:** Hirohito Mori, Masaya Okada, Masatoshi Kanda, Yasunori Yamamoto, Teruki Miyake, Yoichi Hiasa

**Affiliations:** 1 Department of Advanced and Innovative Endoscopy, Ehime University Graduate School of Medicine, Toon, Japan; 2 Department of Gastroenterology, Ehime Rosai Hospital, Niihama, Japan; 3 Endoscopy Center, Ehime University Hospital, Toon, Japan; 4 38050 Department of Gastroenterology and Metabology, Ehime University Graduate School of Medicine, Toon, Japan

Antireflux mucosectomy and antireflux mucosal ablation of treatments for proton pump inhibitor (PPI)-resistant reflux esophagitis have been reported 1 . However, these procedures sometimes result in inaccurate antireflux effect and stricture formation during ulcer healing 2 . To perform more precise treatments with regard to the ulcer healing process and achieving accurate fundoplication, we conducted novel endoscopic suturing ligation and fundoplication (ELF) using a full-thickness endoscopic suturing device (Zeosuture M; Zeon Medical Co., Tokyo, Japan) ( Fig. 1 **a** ) 3 4 .

**Fig. 1:**
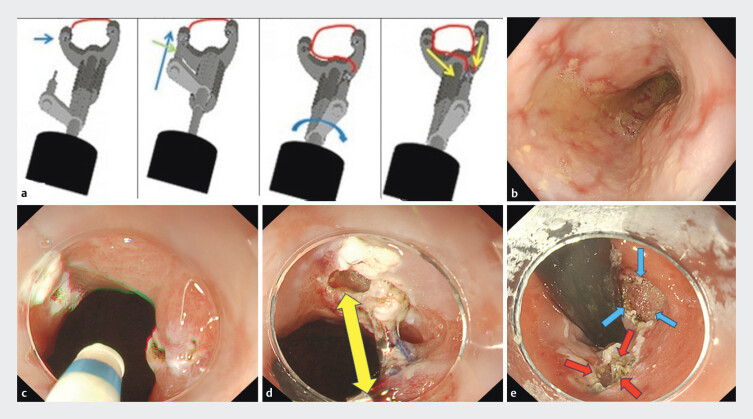
Operation procedure using Zeosuture M (Zeon Medical Co., Tokyo, Japan) and creation of a small artificial ulcer. **a** Procedure for suturing using the Zeosuture M. The suture thread is preloaded onto the front arm of the device (blue arrow). The tissue is grasped between the front arm and rear arm (green arrow). This allows the suture thread to pass through the left side of the tissue in a full-thickness manner. The rear arm is then rotated and repositioned to the right side (blue curved arrow), where the suture thread is similarly passed through the tissue on that side. A ligation device is used to complete the single full-thickness stitch (yellow arrow). **b** Esophagogastroduodenoscopy revealed severe mucosal breaks throughout the esophagus. **c** Markings on anterior and posterior walls of the esophagogastric junction were made under forward and retroflex views. **d** A small 3-mm ring-threaded clip was first placed on the anterior wall. The ring was pulled using another clip, which was placed on the posterior wall, creating mucosal elevation without submucosal injection (yellow arrow). **e** This technique allowed for safe and smaller artificial mucosal defects (5 mm in diameter).

A 73-year-old man who previously underwent distal gastrectomy had been treated for severe reflux esophagitis (Los Angeles Classification grade C) with PPI over 8 years. Esophagogastroduodenoscopy (EGD) revealed mucosal breaks throughout the esophagus ( Fig. 1 **b** , Video 1 ).

The technique of endoscopic suturing ligation and fundoplication for proton pump inhibitor-resistant severe reflux esophagitis using an endoscopic suturing device.Video 1

Markings on anterior and posterior walls of the esophagogastric junction (EGJ) were made under forward and retroflex views ( Fig. 1 **c** ). A small 3-mm ring-threaded clip was first placed on the anterior wall. The ring was pulled using another clip, which was placed on the posterior wall, creating mucosal elevation without submucosal injection ( Fig. 1 **d** ). This technique allowed for safe and small artificial mucosal defects (5 mm in diameter) ( Fig. 1 **e** ). Zeosuture M was attached to the endoscope. The front arm was inserted on the oral side of the mucosal defect, while the second arm with a suturing needle was passed through the mucosal defect from the anal side. A single suture was conducted, compressing the mucosal defect. The suturing thread was tied ( Fig. 2 **a** ). The hernia orifice, which was twice the diameter of the endoscope was reduced after suturing. The endoscope could pass through the EGJ orifice ( Fig. 2 **b** ).

**Fig. 2:**
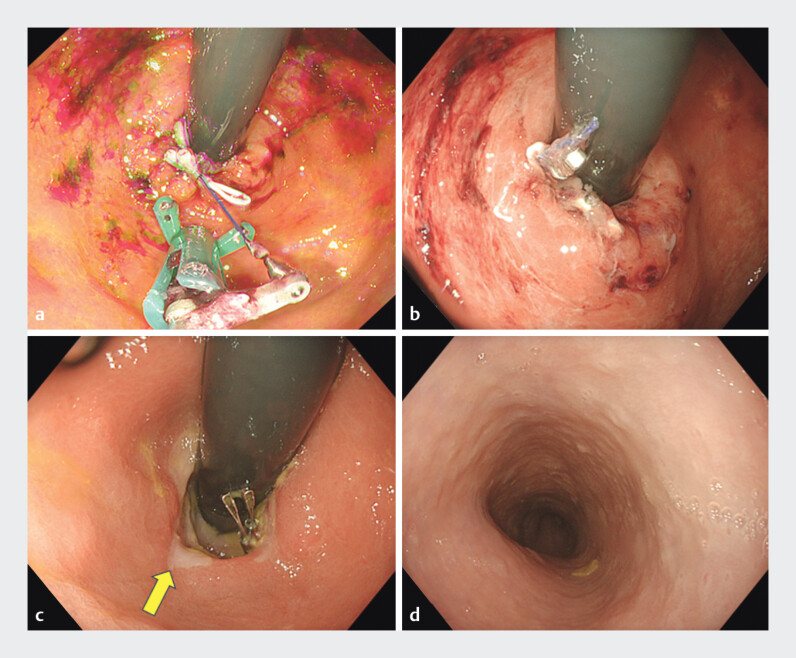
Suturing by Zeosuture M and 30 days after endoscopic fundoplication. **a** A single suture was conducted, compressing the mucosal defect. The suturing thread was tied. **b** The hernia orifice, which was twice the diameter of the endoscope, was reduced after suturing. The endoscope could pass through the esophagogastric junction (EGJ) orifice. **c** Esophagogastroduodenoscopy on postoperative Day 30 revealed the ulcer scar at the EGJ (yellow arrow). **d** The severe mucosal breaks in the esophagus had completely healed after endoscopic suturing ligation and fundoplication.

PPI treatment was suspended after ELF. EGD on postoperative Day 30 revealed the ulcer scar at the EGJ ( Fig. 2 **c** ). Mucosal breaks in the esophagus had completely healed ( Fig. 2 **d** ). The patient remained symptom free with no esophageal stricture.

This case demonstrates successful ELF for PPI-resistant reflux esophagitis, and offers promising therapy for patients with regard to conducting more accurate fundoplication.

Endoscopy_UCTN_Code_TTT_1AO_2AJ

## Notes

Endoscopy E-Videos https://eref.thieme.de/e-videos
*E-Videos* is an open access online section of the journal *Endoscopy* , reporting on interesting cases and new techniques in gastroenterological endoscopy. All papers include a high-quality video and are published with a Creative Commons CC-BY license. Endoscopy E-Videos qualify for HINARI discounts and waivers and eligibility is automatically checked during the submission process. We grant 100% waivers to articles whose corresponding authors are based in Group A countries and 50% waivers to those who are based in Group B countries as classified by Research4Life (see: https://www.research4life.org/access/eligibility/ ). This section has its own submission website at https://mc.manuscriptcentral.com/e-videos .
